# Supplementary material for: Valuing selected WAItE health states using the Time Trade-Off methodology: findings from an online interviewer-assisted remote survey
Source: J Patient Rep Outcomes. 2024 Jan 12;8:6. doi: 10.1186/s41687-023-00674-9 (PMC10786771; doi:10.1186/s41687-023-00674-9)
Supplement: Supplementary file 1 — Supplementary Material 1 [file 41687_2023_674_MOESM1_ESM.docx]

| **Appendix 1 – WAItE Health States Presented** | | | | |
| --- | --- | --- | --- | --- |
| **WAItE Dimension** | **Health State A (2212122)** | **Health State B (2234442)** | **Health State C (4445555)** | **PITS Health State (5555555)** |
| Tired | I almost never get tired | I almost never get tired | I often get tired | I always get tired |
| Walking | I almost never struggle to keep up when I am walking around with others | I almost never struggle to keep up when I am walking around with others | I often struggle to keep up when I am walking around with others | I always struggle to keep up when I am walking around with others |
| Sports | I never avoid doing sports | I sometimes avoid doing sports | I often avoid doing sports | I always avoid doing sports |
| Concentration | I almost never struggle to concentrate on my work/studies | I often struggle to concentrate on my work/studies | I always struggle to concentrate on my work/studies | I always struggle to concentrate on my work/studies |
| Embarrassment | I never feel embarrassed shopping for clothes | I often feel embarrassed shopping for clothes | I always feel embarrassed shopping for clothes | I always feel embarrassed shopping for clothes |
| Unhappy | I almost never feel unhappy because I am unable to do the same things as others | I often feel unhappy because I am unable to do the same things as others | I always feel unhappy because I am unable to do the same things as others | I always feel unhappy because I am unable to do the same things as others |
| Treated Differently | People almost never treat me differently when I go out | People almost never treat me differently when I go out | People always treat me differently when I go out | People always treat me differently when I go out |
