# Supplementary material for: Valuing selected WAItE health states using the Time Trade-Off methodology: findings from an online interviewer-assisted remote survey
Source: J Patient Rep Outcomes. 2024 Jan 12;8:6. doi: 10.1186/s41687-023-00674-9 (PMC10786771; doi:10.1186/s41687-023-00674-9)
Supplement: Supplementary file 2 — Supplementary Material 2 [file 41687_2023_674_MOESM2_ESM.docx]

| **Appendix 2 – Outcome of Interview Invitations** | |
| --- | --- |
| **Outcome** | **n (%)** |
| Invitation for online interview sent via email | 102 (100%) |
| Completed online interview | 35 (34%) |
| Interviewee did not attend scheduled online interview | 3 (3%) |
| Potential interviewee did not want to take part in online interview | 4 (4%) |
| Email address was not recognised | 9 (9%) |
| No response | 51 (50%) |
