# Supplementary material for: Valuing selected WAItE health states using the Time Trade-Off methodology: findings from an online interviewer-assisted remote survey
Source: J Patient Rep Outcomes. 2024 Jan 12;8:6. doi: 10.1186/s41687-023-00674-9 (PMC10786771; doi:10.1186/s41687-023-00674-9)
Supplement: Supplementary file 3 — Supplementary Material 3 [file 41687_2023_674_MOESM3_ESM.docx]

| **Appendix 3 – Characteristics of Respondents to the Initial Screening Survey and Online Interview (Sample 1)** | | | | | | | | |
| --- | --- | --- | --- | --- | --- | --- | --- | --- |
| **Sample** | **Completed Screening Survey (n=102)** | | **Did Not Take Part in Online Interview (n=67)** | | **Completed Online Interview (n=35)** | | **P Value of Difference** | |
| **Gender** | | | | | | | | |
| Male | 51 (50%) | | | 31 (46%) | | 20 (57%) | | 0.477 |
| Female | 50 (49%) | | | 35 (52%) | | 15 (43%) | |  |
| Non-Binary  /Other | 1 (1%) | | | 1 (2%) | | 0 (0%) | |  |
| **Age Band** | | | | | | | | |
| 18-24 | | 16 (16%) | | 14 (21%) | | 2 (6%) | | 0.061* |
| 25-34 | | 17 (17%) | | 7 (10%) | | 10 (29%) | |  |
| 35-44 | | 20 (20%) | | 14 (21%) | | 6 (17%) | |  |
| 45-54 | | 18 (18%) | | 13 (19%) | | 5 (14%) | |  |
| 55-64 | | 16 (16%) | | 8 (12%) | | 8 (23%) | |  |
| 65+ | | 15 (15%) | | 11 (16%) | | 4 (11%) | |  |
| **Income band** | | | | | | | | |
| <£18,800 | | 19 (19%) | | 17 (25%) | | 2 (6%) | | 0.122 |
| £18,801 - £27,162 | | 27 (26%) | | 18 (27%) | | 9 (26%) | |  |
| £27,163 - £36,731 | | 9 (9%) | | 4 (6%) | | 5 (14%) | |  |
| £36,732 - £50,798 | | 12 (12%) | | 6 (9%) | | 6 (17%) | |  |
| > £50,799 | | 27 (26%) | | 16 (24%) | | 11 (31%) | |  |
| Prefer Not To Say | | 8 (8%) | | 6 (9%) | | 2 (6%) | |  |
| **Ethnicity** | | | | | | | | |
| White | | 90 (88%) | | 59 (88%) | | 31 (89%) | | 0.291 |
| Mixed | | 4 (4%) | | 1 (2%) | | 3 (9%) | |  |
| Asian | | 5 (5%) | | 4 (6%) | | 1 (3%) | |  |
| Mixed | | 2 (2%) | | 2 (3%) | | 0 (0%) | |  |
| Other/Prefer Not To Say | | 1 (1%) | | 1 (2%) | | 0 (0%) | |  |
| **Region** | | | | | | | | |
| East Anglia | | 9 (9%) | | 8 (12%) | | 1 (3%) | | 0.477 |
| East Midlands | | 8 (8%) | | 7 (10%) | | 1 (3%) | |  |
| London | | 15 (15%) | | 11 (16%) | | 4 (11%) | |  |
| North West | | 14 (14%) | | 7 (10%) | | 7 (20%) | |  |
| Northern Ireland | | 3 (3%) | | 2 (3%) | | 1 (3%) | |  |
| Scotland | | 10 (10%) | | 5 (7%) | | 5 (14%) | |  |
| South East | | 12 (12%) | | 9 (13%) | | 3 (9%) | |  |
| South West | | 7 (7%) | | 4 (6%) | | 3 (9%) | |  |
| Wales | | 6 (6%) | | 3 (4%) | | 3 (9%) | |  |
| West Midlands | | 10 (10%) | | 5 (7%) | | 5 (14%) | |  |
| Yorkshire & Humberside | | 8 (8%) | | 6 (9%) | | 2 (6%) | |  |
| **Employment** | | | | | | | | |
| Paid Employment | | 52 (51%) | | 32 (48%) | | 20 (57%) | | 0.544 |
| Self-Employed | | 8 (8%) | | 6 (9%) | | 2 (6%) | |  |
| Unemployed | | 8 (8%) | | 6 (9%) | | 2 (6%) | |  |
| Full-Time Student | | 5 (5%) | | 4 (6%) | | 1 (3%) | |  |
| Looking After Home / Family | | 10 (10%) | | 8 (12%) | | 2 (6%) | |  |
| Retired | | 16 (16%) | | 9 (13%) | | 7 (20%) | |  |
| Other | | 1 (1%) | | 0 (0%) | | 1 (3%) | |  |
| **Highest Educational Qualification** | | | | | | | | |
| Degree or Equivalent | | 47 (46%) | | 25 (37%) | | 22 (63%) | | 0.257 |
| Higher Education Below Degree | | 12 (12%) | | 10 (14%) | | 2 (6%) | |  |
| A-Level/AS-Level | | 21 (21%) | | 17 (25%) | | 4 (11%) | |  |
| GCSE Grade A* - C | | 13 (13%) | | 9 (13%) | | 4 (11%) | |  |
| GCSE Grade D – G | | 3 (3%) | | 2 (3%) | | 1 (3%) | |  |
| Other | | 1 (1%) | | 1 (2%) | | 0 (0%) | |  |
| No Formal Qualifications | | 5 (5%) | | 3 (4%) | | 2 (6%) | |  |
| **Weight Status** | | | | | | | | |
| Underweight | | 4 (4%) | | 2 (3%) | | 2 (6%) | | 0.648 |
| Normal/Healthy Weight | | 56 (55%) | | 37 (55%) | | 19 (54%) | |  |
| Overweight | | 38 (37%) | | 26 (39%) | | 12 (34%) | |  |
| Obese | | 3 (3%) | | 2 (3%) | | 1 (3%) | |  |
| Prefer Not To Say | | 1 (1%) | | 0 (0%) | | 1 (3%) | |  |
| ˆDifferences between the samples who did and did not complete the online interview measured using Chi-Squared tests.*** Statistically significant at 1% level. ** Statistically significant at 5% level. * Statistically significant at 10% level. | | | | | | | | |
