# Supplementary material for: Valuing selected WAItE health states using the Time Trade-Off methodology: findings from an online interviewer-assisted remote survey
Source: J Patient Rep Outcomes. 2024 Jan 12;8:6. doi: 10.1186/s41687-023-00674-9 (PMC10786771; doi:10.1186/s41687-023-00674-9)
Supplement: Supplementary file 4 — Supplementary Material 4 [file 41687_2023_674_MOESM4_ESM.docx]

| **Appendix 4 – TTO and VAS Values for WAItE Health States (By Sample)** | | | | |
| --- | --- | --- | --- | --- |
| **Full Estimation Sample (n=42)** | | | | |
| **Health State** | **Health State A (2212122)** | **Health State B (2234442)** | **Health State C (4445555)** | **PITS State (5555555)** |
| TTO Mean (SD) | 0.95 (0.09) | 0.79 (0.19) | 0.39 (0.48) | 0.23 (0.54) |
| TTO Median (IQR) | 1 (0.95 – 1) | 0.80 (0.70 – 0.95) | 0.50 (0.20 – 0.70) | 0.33 (0.05 – 0.60) |
| Valuing State WTD (%) | 0 (0%) | 0 (0%) | 4 (10%) | 7 (17%) |
| VAS Mean (SD) | 84.48 (11.39) | 59.31 (12.89) | 28.45 (15) | 11.50 (11.78) |
| VAS Median (IQR) | 87.50 (80 – 90) | 60 (50 – 65) | 25.50 (20 – 40) | 10 (0 – 20) |
| **Sample 1 (n=35)** | | | | |
| **Health State** | **Health State A (2212122)** | **Health State B (2234442)** | **Health State C (4445555)** | **PITS State (5555555)** |
| TTO Mean (SD) | 0.94 (0.10) | 0.82 (0.19) | 0.41 (0.47) | 0.23 (0.55) |
| TTO Median (IQR) | 0.99 (0.95 – 1) | 0.85 (0.70 – 1) | 0.50 (0.20 – 0.75) | 0.30 (0.05 – 0.60) |
| Valuing State WTD (%) | 0 (0%) | 0 (0%) | 3 (9%) | 6 (17%) |
| VAS Mean (SD) | 84.66 (12.10) | 60.74 (13.05) | 18.57 (8.52) | 12.94 (12.22) |
| VAS Median (IQR) | 90 (80 – 90) | 60 (50 – 70) | 20 (10 – 20) | 10 (1 – 20) |
| **Sample 2 (n=7)** | | | | |
| TTO Mean (SD) | 0.99 (0.02) | 0.69 (0.19) | 0.25 (0.51) | 0.21 (0.56) |
| TTO Median (IQR) | 1 (0.95 – 1) | 0.75 (0.60 – 0.80) | 0.45 (0 – 0.55) | 0.40 (0 – 0.60) |
| Valuing State WTD (%) | 0 (0%) | 0 (0%) | 1 (14%) | 1 (14%) |
| VAS Mean (SD) | 83.57 (7.48) | 52.14 (9.94) | 0.23 (0.51) | 4.29 (5.34) |
| VAS Median (IQR) | 85 (80 – 90) | 50 (40 – 60) | 0.45 (0 – 0.55) | 0 (0 – 10) |
