# Supplementary material for: Valuing selected WAItE health states using the Time Trade-Off methodology: findings from an online interviewer-assisted remote survey
Source: J Patient Rep Outcomes. 2024 Jan 12;8:6. doi: 10.1186/s41687-023-00674-9 (PMC10786771; doi:10.1186/s41687-023-00674-9)
Supplement: Supplementary file 5 — Supplementary Material 5 [file 41687_2023_674_MOESM5_ESM.docx]

**Valuing Selected WAItE Health States using the Time Trade-Off Methodology: Findings from an Online Interviewer-Assisted Remote Survey**

**Abstract**

**Purpose:** The Weight-Specific Adolescent Instrument for Economic Evaluation (WAItE) is a physical weight-specific patient reported outcome measure for use in adolescence. The purpose of this study was to use the Time Trade-Off (TTO) methodology, administered using an online interviewer-assisted remote survey, to obtain utility values for several health states from the WAItE descriptive system from a sample of the UK adult general population.

**Methods:** The adult sample was gathered using a market research company and a sample of local residents. All participants completed the same interviewer-assisted remote survey, which included rating WAItE states of varying impairment using the TTO.

**Results:** 42 adults completed the survey. Utility values were gathered for four health states, ranging from low impairment to the most severe health from the WAItE descriptive system (the Pits state). Consistent orderings of the WAItE health states were observed; the health state with the lowest level of impairment was valued highest and the Pits state was valued lowest. Several respondents (n=7, 17%) considered the Pits state to be worse than death; however, the mean value of this health state was 0.23.

**Conclusions:** The utility value of the Pits state relative to death generated from this study will be used to anchor latent values for WAItE health states generated from a Discrete Choice Experiment onto the 0=death, 1 =full health Quality Adjusted Life Year (QALY) scale as part of a valuation study for the WAItE in the UK population. This study also provides further evidence that interviewer-assisted digital studies are feasible for collecting TTO data.

1. **Background**

The prevalence of obesity in young people is a huge public health concern in the United Kingdom (UK). Although there have been efforts to curtail the rising prevalence of obesity, a World Health Organisation (WHO) report estimated that a third of adolescents in Europe are overweight or obese [1]. Additionally, the UK societal costs of overweight and obesity are predicted to reach £49.9 billion per year by 2050 [2]. Weight-management interventions targeting obesity in young people are ongoing [3] and are an important strategy to reduce the societal burden of obesity. However, policymakers must make resource allocation decisions based on cost-effectiveness evidence to ensure value for money. Furthermore, there is currently no validated weight-specific HRQoL measure for adolescents that can be used in economic evaluation.

As such, the Weight-Specific Adolescent Instrument for Economic Evaluation (WAItE) has been developed for use in adolescence (ages 11-18), consisting of seven dimensions relating to tiredness, walking, participation in sports, concentration, embarrassment, unhappiness, and being treated differently [4]. Each dimension is expressed using a 5-level frequency response scale with increasing degrees of severity ranging from “never” to “always”. The WAItE descriptive system is available on request.

During development, the WAItE’s psychometric properties were thoroughly examined, and each dimension was informed by a combination of Rasch analysis, psychometric assessment and re-visiting the qualitative material [5]. Additionally, a robust validation of the WAItE has been conducted to provide evidence of its criterion validity and reliability for future use [6]. This involved examination of the concurrent validity of the WAItE in comparison to other validated patient-related HRQoL tools and an assessment of the test-retest reliability of the WAItE to explore its consistency.

Despite being specifically designed to be a preference-based measure, the WAItE currently has no associated value set and therefore cannot be used to generate quality-adjusted life years (QALYs), which are the basis of cost-utility analysis (CUA). To address this, an algorithm was developed which mapped responses from the WAItE to the Child Health Utility 9 Dimension (CHU9D) value set [7]. However, this is considered to be a second-best approach, with the ‘gold standard’ valuation method being direct elicitation of preference values through a valuation study [8].

Given the developmental work already completed on the WAItE, a natural progression is to develop a preference algorithm to generate a set of preference values for the WAItE which are based on direct elicitation of preferences from a valuation study. A discrete choice experiment (DCE) study (a method of eliciting preference by asking participants to make a choice between two or more alternatives) is ongoing to develop a value set for the WAItE classification system [9]. The DCE will be delivered to members of the adult general population of the UK using an online survey. There were several reasons for the decision, to use an adult sample, including the fact that adults may have a greater capacity to understand complex preference elicitation tasks. Furthermore, as adult preferences are typically used to generate value sets for adult preference-based measures, using adult preferences to value adolescent preference-based measures provides a comparability in the methods used to value health states for both adolescents and adults. The choice of whose preferences to use in the valuation of child and adolescent health states is a matter of normative debate, and our choice is further discussed in the study protocol [9].

A DCE alone is not sufficient to generate a set of preference values, as the results are interpreted on a latent scale rather than the 0=death, 1=full health QALY scale. There are a number of options for converting the DCE results onto the 0=death, 1=full health QALY scale, and currently there is no standard method of anchoring [10].

One method that has previously been used to anchor latent DCE results onto the 0=death, 1=full health QALY scale is a standalone TTO study. This anchoring method has been successfully used in both Australia and China [11, 12] to convert DCE results for the CHU-9D on the latent scale to the 0=death, 1=full health QALY scale. By obtaining a value for the lowest WAItE state (the Pits state) relative to death, the latent coefficients obtained in the DCE will be reweighted on the 0=death, 1=full health QALY scale by ensuring that 0 represents death, therefore providing the WAItE with an appropriate preference-based value set for use in CUA.

The TTO technique developed by Torrance and colleagues [13], presents a simple and intuitive alternative to ensure that health state values are anchored with 0 representing death. This technique presents respondents with two alternative “lives”, either a “life” in full health or a “life” in an impaired health state (both followed by death), and respondents are asked to identify a time spent in full health in which they would consider that “life” to be equivalent to spending a relatively longer, or equal, but fixed amount of time in the impaired health state “life” [14].

One limitation the standard TTO methodology presents is the evaluation of states considered worse than being dead. In the standard TTO values are bound between 1 and 0, and no time amount of time can be given up from the full health “life” to avoid the impaired health state that would generate a negative utility value (that can be associated with worse than being dead). To enable states considered better than dead (BTD) and states worse than dead (WTD) to be valued as part of the same valuation exercise, the composite TTO (cTTO) has been developed [15]. The cTTO uses the standard TTO for BTD health states and the ‘lead-time’ TTO [16] for WTD health states. The lead-time TTO involves giving the respondent a fixed and equal amount of extra time spent in full health to the beginning of both “lives”. Thus, the total length of each live remains equal, and the time spent in the impaired health state also remains fixed and equal to that of the standard TTO, yet the available time in full health that can be given up to avoid the impaired health state is now greater than the time in the impaired health state. Implicitly, this means that health states that are considered WTD can generate negative utility values that are comparable with the positive BTD health state values.

As TTO is traditionally an interviewer-led method of preference elicitation, the use of videoconferencing software for delivering interviews has become an important consideration, particularly when external factors prevent traditional face-to-face interviews being delivered. It has been shown that with several changes to the recruitment and interview process, TTO interviews using videoconferencing software are feasible and yield similar results to traditional face-to-face interviews [17, 18, 19].

The principal aim of this study was to use the TTO method to obtain a utility value for the WAItE Pits state relative to death, to then anchor the latent coefficients generated from a DCE obtained as part of a UK valuation of the WAItE instrument on to the 0=death, 1=full health QALY scale. This will enable QALYs to be directly generated from the WAItE for use in CUA~~.~~ A secondary aim of the study was to assess the feasibility of the use of an online modality of delivering TTO interviews.

1. **Methods**

**Ethics**

Ethical approval was granted by Newcastle University’s Faculty of Medical Sciences Ethics Board (Reference Number 9978/2020).

**Survey Development**

A bespoke cTTO survey was designed using the Qualtrics software package [20]. In line with the DCE part of the full valuation study, the cTTO was designed to be completed by a sample of the UK adult general population. the TTO methodology is considered overly cognitively demanding for children and adolescents, and ethical concerns have been raised about using techniques that involve consideration of death with children and adolescents [21].

The main part of the TTO survey was structured as follows. First, participants were asked to complete the WAItE for themselves to familiarise themselves with the wording, formatting, and descriptive system of the questionnaire. The participants were then asked to read aloud four health states generated from the WAItE descriptive system and rank them from their most preferred to least preferred, including the Pits State, which is defined by the worst level of each dimension. Aside from the Pits state, three WAItE states were chosen to represent ‘mild impairment’, ‘moderate impairment’ and ‘severe impairment’. The health states presented to the participants are shown in Appendix 1. The respondents were then asked to score each of the WAItE health states on a scale from 0 (‘the worst health you can imagine’) to 100 (‘the best health you can imagine’) using a visual analogue scale (VAS). Before scoring each health state, they were reminded of what position they had ranked the health state in the previous section.

To familiarise themselves with the format and wording of the TTO, in the next section of the interview, the respondents completed two practice TTO tasks. They were first asked to value being ‘In a wheelchair’ and then ‘The worst health state you can imagine’. The inclusion of practice profiles is standard practice in TTO studies, as it is argued that their inclusion improves the participants’ understanding of the exercise and improves data quality [22,23].

The respondents then completed the TTO tasks, valuing the moderate impairment state first, the severe impairment state, the mild impairment state, and finally the Pits state. In line with the valuation protocols for the various versions of the EQ-5D [24-26], a cTTO was used, with the respondents presented with a standard TTO to value health states BTD and a lead-time TTO for any health states they considered WTD. In line with the various EQ-5D protocols, there was a 10-year fixed duration for the impaired health state “life” in the standard TTO (BTD health states) and a 20-year duration in the lead-time TTO “life” (WTD health states), with 10 years of full-health followed by 10 years of impaired health in that sequence [15, 24]. The iterative procedure followed a ‘ping pong approach’ [27], with the length of the time in full health varied until the respondent was indifferent between the two “lives” (full health vs 10-years in the impaired health state). Respondents were able to reach indifference at a minimum of half year increments.

In the final part of the interview, the respondents completed three post-survey questions, related to their understanding of the survey, their ease in telling the difference between different health states and their difficulty in deciding on their answers.

**Piloting**

Following initial survey testing with a convenience sample of Newcastle University colleagues not familiar with the TTO methodology, a round of external pilot testing of the TTO survey was conducted using a convenience sample recruited from a local community group in the North-East of England. The opportunity to pilot the survey was advertised to the group via social media. Individuals responded to the advertisement and were sent a copy of the study information sheet to read before consenting to take part. Pilot interviews were completed by two trained interviewers in August 2021 on the videoconferencing platform Zoom [28], and each respondent received a £15 shopping voucher as compensation for their time. Piloting via the community group enabled the survey to be tested on a range of genders, ages, and backgrounds to provide variation in our pilot sample. The interview script (which was based on the valuation protocols for the various versions of the EQ-5D [24-26]) was followed for each interview to ensure consistency and respondent understanding and to mitigate interviewer bias. No changes were made to the script or cTTO procedure following piloting, so the pilot responses were included in the full estimation sample.

**Recruitment and Sampling**

The main study sample was gathered with the assistance of the market research company *Dynata* [29]*.* To gather a balanced sample of adults from the general population, potential respondents first completed a screening survey. In this screening survey, sociodemographic information was collected including gender, age band, ethnicity, region, income band, employment status, highest educational qualification, and self-reported weight status. At the end of the screening survey, the participants consented to be contacted via email to take part in the online TTO interview and stated their availability for interview. Quotas implemented by *Dynata* ensured that this sample was nationally representative in terms of gender, age band, and geographical location. Those respondents who reported being from the North-East of England were excluded from this sample to avoid over-representation because the pilot sample was exclusively sampled from this geographical area. Our overall target sample size was 40, like previous studies that have conducted a standalone TTO for the purposes of anchoring the latent coefficients from a DCE in the context of child health [10,11].

**Interview Procedure**

Prior to the interview, the participants were sent a meeting link via email along with a comprehensive participant information sheet which they were asked to read prior to the interview. As with the pilot interviews, an interview script was followed by the trained interviewers to ensure consistency and mitigate interviewer bias. The interviewer shared their screen for the duration of the interview, allowing the respondent to see the online survey on their screen whilst also being able to converse with the interviewer. After being introduced to the survey, having the opportunity to ask any questions related to the participant information sheet and verbally consenting to take part in the online interview, the main part of the survey began, as detailed in the ‘Survey Development’ sub-section. At the conclusion of the survey, the respondents were thanked for their participation in the interview and the interview was ended. Each participant was paid the equivalent of £15 in either panel points or shopping vouchers as a thank you for their time completing the interview.

**Data Analysis**

For those states considered BTD, the TTO utility scores were calculated as: $x$/10, with $x$ representing the number of years at which the respondent was indifferent between the time spent in full health and 10 years in the WAItE health state in question. For those states considered WTD, the TTO utility scores were calculated as ($x$ – 10)/10, bounding these utilities between -1 and 0. Descriptive summary statistics for the responses to the TTO and VAS were calculated, including the mean, median, standard deviation, and inter-quartile range. The responses to the WAItE were converted to a WAItE total sum score, scored between 7 (the best possible health state) and 35 (the worst possible health state). The participant’s sociodemographic characteristics from the screening survey and their responses to the post-survey questions were presented as frequencies and percentages. The two sets of data were linked using a personalised identifying code. Data were analysed using Stata version 16.0 [30].

1. **Results**

In the pilot sample, 14 individuals responded to the advertisement, and 7 pilot interviews were completed. In the main sample, 102 adults who completed the initial screening survey were invited to participate in the study via email. Of the potential participants invited to take part in the online interview, 9 (9%) could not be contacted, and a further 5 (50%) did not respond to the emails asking them to participate in the interview (see Appendix 2). Of those who responded to the email, 4 (4%) declined the invitation, and 3 participants (3%) did not attend. This gave a final sample size of 35 in the main sample.

Combining the pilot sample and the main sample (hereafter the ‘full estimation sample’) gave a final sample size of 42, in line with our target sample size of 40. All participants fully completed the online interview. Table 1 shows the socio-demographic characteristics of the estimation sample. The full estimation sample was 55% male, 88% white and the modal age category was 25-34 (31%). The sample was relatively evenly spread across the geographical regions of the UK. Most of the participants were either in paid employment or self-employed (62%), and 62% of participants had a degree. The majority (60%) of the participants self-reported as being a normal/healthy weight, with 31% reporting being overweight and 2% being obese.

**[TABLE 1]**

Table 2 shows the responses to the WAItE. Overall, the respondents reported being in relatively good health, with the modal answer being ‘never’ (the highest level in the WAItE classification system) for five of the seven categories. The exceptions to these were the attributes related to tiredness and concentration. One respondent reported themselves as being in full health, corresponding to the highest level in each of the WAItE attributes.

**[TABLE 2]**

Table 3 shows the mean (median) values from the TTO and VAS. The mild impairment state was valued the highest 0.95 (1), followed by the moderate impairment state 0.79 (0.80), the severe impairment state 0.39 (0.50) and the Pits State 0.23 (0.33). While no participants valued Health State A or Health State B WTD, 4 participants (10%) valued Health State C WTD, and 7 participants (17%) valued the Pits State WTD. The mean (median) VAS value of Health State A was 85 (88), the value of Health State B was 59 (60), the value of Health State C was 28 (26) and the Pits state was 12 (10).

**[TABLE 3]**

Most of the participants (98%) strongly agreed or agreed that it was easy to understand the questions in the online interview. Similarly, 95% strongly agreed or agreed that it was easy to tell the difference between the health states presented in the online interview. 45% of the participants strongly agreed or agreed that it was difficult to decide on their answers, while 41% of the participants strongly disagreed or disagreed and 14% neither agreed nor disagreed.

1. **Discussion**

**WAItE Utility Values**

The mean and median values from the TTO followed the pattern one would expect *a priori*, with the mild impairment state being valued the highest (0.950), followed by the moderate impairment state (0.794), the severe impairment (0.386) and finally the Pits State (0.229). As one may expect given the small sample size and nature of the task, there are large standard deviations around the mean TTO value of both Health State C (0.48) and the PitsState (0.54), indicating a significant level of individual level heterogeneity. Previous work has also found an increased level of heterogeneity in the valuation of more severe states when using the cTTO [15]. One reason for this could be the fact that the valuation space for the lead-time TTO (which is more likely to be used for severe health states) is larger (-1 to 1) than that of the standard TTO (0-1). It also could be due to the increased complexity of the lead-time TTO. The mean and median values for the VAS also follow the pattern one would expect *a priori*, with a logical ordering of the health states identical to the TTO responses.

The mean TTO values of the Pits State will be used to anchor the latent estimates from an ongoing DCE study (adult sample, N=1,005) to provide a scoring algorithm for the WAItE for the UK population, by re-scaling these latent estimates onto the 0=death, 1=full health QALY scale needed for CUA. This will allow for the calculation of weight-specific QALYs in the adolescent population.

**Using Interviewer Assisted Digital TTO Surveys**

As well as contributing to the literature regarding the use of standalone TTO studies as a method of anchoring in valuation studies, this study has also provided further evidence that it is feasible to collect TTO data to an appropriate standard using digital interviews. Although in person TTO interviews have traditionally been the most common used method (although not necessarily seen as the “gold standard”[18]), when there are limited resources available (both human and financial) or where physical barriers or external factors exist, digital methods appear to be an acceptable and feasible alternative.

Related to this, it is worthwhile discussing the findings from this study in relation to the points raised by Lipman [17] with regards to the advantages, disadvantages and lessons learnt from interviewer assisted digital TTO interviews. As noted by Lipman [17], there could be a higher chance of respondents cancelling on short notice or not showing up at all when conducting interviewer-assisted remote interviews. In this study, only three respondents did not show up to their online interview, even without the use of reminder emails. As further noted by Lipman [17], there is a possibility that the use of interviewer assisted digital TTO interviews may introduce selection bias, where respondents with certain sociodemographic characteristics are more likely to take part in the interview. There is some evidence of selection bias in this study. As shown in Appendix 3, there are some differences between the characteristics of individuals who completed the screening survey (which was nationally representative in terms of gender, age band, and geographic area) but did not take part in the online interview, and those individuals who completed the online interview. For instance, those who completed the online interview were less likely to be in the lowest age category (18-24), less likely to be in the lowest income category (<£18,800) and more likely to have a degree level education. However, these differences can be considered relatively small.

**Strengths and Limitations**

There are several strengths to the study. Firstly, all participants who started the online TTO interview fully completed the interview, indicating that the online interview, and interview process more generally, was fit for purpose. Furthermore, most of the participants indicated that the questions in the survey were easy to understand and that it was easy to tell the difference between health states presented. Although 45% of respondents agreed that it was difficult to decide on their answers, it should be noted that the TTO is a cognitively complex task which requires the careful consideration of health status and time preference, and therefore some level of difficulty is to be expected. It is again worth noting that the mean and median values for the TTO followed a logical pattern that one would expect *a priori*, further indicating that the online interview process was fit for purpose.

However, there are also several limitations to this study that should be considered when interpreting the findings. Firstly, the size of the full estimation sample (n=42) is low compared with other patient preference studies. However, as stated previously, this sample size is comparable to several other studies in the literature that have conducted a standalone TTO for the purposes of anchoring the latent coefficients from a DCE in the context of child health [10,11], both of which had a final sample size of 38. Furthermore, the final sample itself was composed of a larger sample collected through a market research company (n=35), and a sample gathered from the local area (n=7). Although the main online survey completed by the participants was identical, the method of collecting the sociodemographic data was slightly different between the two samples, and the two samples of data were collected at different points in time. However, as shown in Appendix 4 the answers to the online interview were very similar between the two samples. Excluding the pilot responses from the full estimation sample made very little difference to the overall results and interpretation of the findings.

Secondly, the TTO survey was completed by a sample of adults rather than adolescents. These valuations from adults may be different to those from adolescents. As previously noted, the choice of whose preferences to use is a normative debate, and there is currently limited guidance on the most appropriate methods to use [31]. Planned future research will investigate whether responses in preference elicitation tasks in the context of the WAItE are comparable between adults and adolescents.

Thirdly, although every effort was made to ensure that the sample was representative of the UK adult population, the sample is slightly unbalanced in some demographic characteristics, including age band, income band, and self-reported weight status. Given the relatively small sample size, the likelihood of an imbalance was expected to be high due to sampling uncertainty.

Fourthly, due to the COVID-19 pandemic, the interviews took place online rather than face-to-face as originally planned. This meant that some of the contextual factors that would be controlled for in an in-person setting could not be addressed. Another consequence of the use of online surveys is that the electronic devices used by the participants may have had heterogeneous size screens, meaning that the VAS presented to the participants may have been displayed in different lengths, which could influence the participant’s response to this task. Finally, there are limitations with the TTO methodology. For instance, the QALY approach assumes that the utility estimates generated are independent from the length of time presented in the questionnaire, and therefore the length of time spent in the impaired health state presented to the respondents may impact the results obtained. The cTTO technique further relies on this assumption as WTD tasks change the duration of the impaired health state by adding extra time in full health. Moreover, the duration of the time spent in full health relative to impaired health and the sequence in which they are presented can introduce new concerns about framing. The literature has advocated for more consistency in the design of TTO and cTTO preference elicitation exercises [15].

1. **Conclusion**

This study used TTO methods to estimate the values of several health states defined by the WAItE descriptive system, a weight-specific patient reported outcome measure for use in adolescence. This included an estimate of the P State, which will be used in an ongoing valuation study of the WAItE in the UK population to anchor the latent coefficients from a DCE study onto the 0=death, 1=full health QALY scale. In addition to the contribution to the literature regarding the valuation of weight-specific HRQoL in adolescence, the study also contributes to the growing literature suggesting that collecting TTO data using an interview-assisted digital survey is a feasible alternative to the traditional face-to-face TTO interviews.

**References**

1. Hernández-Quevedo C, Gauci C, Rechel B (2019) Childhood obesity in Europe and policies to address it. Eurohealth.;25(1):7-10.
2. https://www.gov.uk/government/publications/health-matters-obesity-and-the-food-environment/health-matters-obesity-and-the-food-environment--2#:~:text=The%20overall%20cost%20of%20obesity,%C2%A349.9%20billion%20per%20year.
3. Jones AR, Tovée MJ, Cutler LR, Parkinson KN, Ells LJ, Araujo-Soares V, Pearce MS, Mann KD, Scott D, Harris JM, Adamson AJ (2018) Development of the MapMe intervention body image scales of known weight status for 4–5 and 10–11 year old children. J Public Health. Sep 1;40(3):582-90.
4. Oluboyede Y, Hulme C, Hill A (2017) Development and refinement of the WAItE: a new obesity-specific quality of life measure for adolescents Qual Life Res Aug;26:2025-39.
5. Oluboyede Y, Smith AB, Hill A, Hulme C (2019) The weight-specific adolescent instrument for economic evaluation (WAItE): psychometric evaluation using a Rasch model approach. Qual Life Res. Apr 15;28:969-77.
6. Oluboyede Y, Robinson T (2019) Measuring weight-specific quality of life in adolescents: an examination of the concurrent validity and test-retest reliability of the WAItE. Value in Health. Mar 1;22(3):348-54.
7. Robinson T, Oluboyede Y (2019) Estimating CHU-9D utility scores from the WAItE: a mapping algorithm for economic evaluation. Value in Health. Feb 1;22(2):239-46.
8. Brazier J, Ratcliffe J, Saloman J, Tsuchiya A (2017) Measuring and valuing health benefits for economic evaluation. Oxford University Press;
9. Robinson T, Hill S, Oluboyede Y (2021) Developing a preference-based measure for weight-specific health-related quality of life in adolescence: the WAItE UK valuation study protocol. BMJ Open. Nov 1;11(11):e054203.
10. Webb EJ, O’Dwyer J, Meads D, Kind P, Wright P (2020) Transforming discrete choice experiment latent scale values for EQ-5D-3L using the visual analogue scale. Eur J Health Econ. Jul;21:787-800.
11. Ratcliffe J, Chen G, Stevens K, Bradley S, Couzner L, Brazier J, Sawyer M, Roberts R, Huynh E, Flynn T (2015) Valuing Child Health Utility 9D health states with young adults: insights from a time trade off study. Appl. Health Econ. Health Policy. Oct;13:485-92.
12. Chen G, Xu F, Huynh E, Wang Z, Stevens K, Ratcliffe J (2019) Scoring the Child Health Utility 9D instrument: estimation of a Chinese child and adolescent-specific tariff. Qual Life Res. Jan 15;28:163-76.
13. Torrance GW (1976) Social preferences for health states: an empirical evaluation of three measurement techniques. Socio-Econ. Plan. Sci.. Jan 1;10(3):129-36.
14. Torrance GW (1986) Measurement of health state utilities for economic appraisal: a review. J. Health Econ.. Mar 1;5(1):1-30.
15. Janssen BM, M, Versteegh MM, Stolk EA (2013) Introducing the composite time trade-off: a test of feasibility and face validity. Eur J Health Econ Jul;14:5-13.
16. Robinson A, Spencer A (2006) Exploring challenges to TTO utilities: valuing states worse than dead Health Econ. Apr;15(4):393-402.
17. Lipman SA. Time for tele-TTO? (2021) Lessons learned from digital interviewer-assisted time trade-off data collection. Patient. 2021 Sep;14(5):459-69.
18. Rowen D, Mukuria C, Bray N, Carlton J, Longworth L, Meads D, O'Neill C, Shah K, Yang Y (2022) Assessing the comparative feasibility, acceptability and equivalence of videoconference interviews and face-to-face interviews using the time trade-off technique. Soc. Sci. Med.. Sep 1;309:115227.
19. Estévez-Carrillo A, Dewilde S, Oppe M, Ramos-Goñi JM (2022) Exploring the comparability of face-to-face versus video conference-based composite time trade-off interviews: insights from EQ-5D-Y-3L valuation studies in Belgium and Spain. Patient. Sep;15(5):521-35.
20. Qualtrics, Provo, UT, USA. <https://www.qualtrics.com>
21. Rogers HJ, Marshman Z, Rodd H, Rowen D (2021) Discrete choice experiments or best-worst scaling? A qualitative study to determine the suitability of preference elicitation tasks in research with children and young people. Patient-Rep. Outcomes.Dec;5:1-1.
22. Dolan P (1997) Modeling valuations for EuroQol health states. Med. Care. Nov 1:1095-108.
23. Shen J, Breckons M, Vale L, Pickard R (2019) Using time trade-off methods to elicit short-term utilities associated with treatments for bulbar urethral stricture. PharmacoEconomics-Open. Dec;3:551-8.
24. Oppe M, Devlin NJ, van Hout B, Krabbe PF, de Charro F (2014) A program of methodological research to arrive at the new international EQ-5D-5L valuation protocol. Value in Health Jun 1;17(4):445-53.
25. Stolk E, Ludwig K, Rand K, van Hout B, Ramos-Goñi JM (2019) Overview, update, and lessons learned from the international EQ-5D-5L valuation work: version 2 of the EQ-5D-5L valuation protocol. Value in Health. Jan 1;22(1):23-30.
26. Ramos-Goñi JM, Oppe M, Stolk E, Shah K, Kreimeier S, Rivero-Arias O, Devlin N (2020) International valuation protocol for the EQ-5D-Y-3L. Pharmacoeconomics. Jul;38:653-63.
27. Attema AE, Edelaar-Peeters Y, Versteegh MM, Stolk EA (2013) Time trade-off: one methodology, different methods. Eur J Health Econ. Jul;14:53-64.
28. Zoom, San Joe, CA, USA. <https://zoom.us/>
29. Dynata, Shelton, CT, USA. <https://www.dynata.com/>
30. StataCorp. (2019) Stata Statistical Software: Release 16. College Station, TX: StataCorp LLC.

**Statements and Declarations**

This study is funded as part of the MapMe2 study. The MapMe2 study is funded by the National Institute for Health and Care Research [NIHR127745] Trial ID: ISRCTN12378125.

**Competing Interests**

The authors have no relevant financial or non-financial interests to disclose.

**Author Contributions**

YO acquired the funding. TR, SH, GOL and YO contributed to the design of the study. SH, GOL, WK and AK carried out the interviews. The first draft of the manuscript was written by TR and all authors commented on previous versions of the manuscript. All authors read and approved the final manuscript.

**Ethics**

The Newcastle University Medical School Ethics Committee approved the study (Reference 9978/2020).

**Consent**

Informed consent was obtained from all individual participants included in the study.

| **Table 1 – Participant Characteristics** | | | |
| --- | --- | --- | --- |
| **Sample** | **Full Sample (n=42)** | **Main Sample**  **(n=35)** | **Pilot Sample**  **(n=7)** |
| **Gender** | |  |  |
| Male | 23 (55%) | 20 (57%) | 3 (43%) |
| Female | 19 (45%) | 15 (43%) | 4 (57%) |
| **Age Band** |  |  |  |
| 18-24 | 3 (7%) | 2 (6%) | 1 (14%) |
| 25-34 | 13 (31%) | 10 (29%) | 3 (43%) |
| 35-44 | 7 (17%) | 6 (17%) | 1 (14%) |
| 45-54 | 5 (12%) | 5 (14%) | 0 (0%) |
| 55-64 | 9 (21%) | 8 (23%) | 1 (14%) |
| 65+ | 5 (12%) | 4 (11%) | 1 (14%) |
| **Income band** |  |  |  |
| <£18,800 | 2 (5%) | 2 (6%) | 1 (14%) |
| £18,801 - £27,162 | 10 (24%) | 9 (26%) | 0 (0%) |
| £27,163 - £36,731 | 5 (12%) | 5 (14%) | 0 (0%) |
| £36,732 - £50,798 | 8 (19%) | 6 (17%) | 1 (14%) |
| > £50,799 | 14 (33%) | 11 (31%) | 3 (43%) |
| Prefer Not To Say | 3 (7%) | 2 (6%) | 1 (14%) |
| **Ethnicity** |  |  |  |
| White | 37 (88%) | 31 (89%) | 6 (86%) |
| Asian | 1 (3%) | 1 (9%) | 0 (0%) |
| Mixed | 3 (7%) | 3 (9%) | 0 (0%) |
| Prefer Not To Say | 1 (2%) | 0 (0%) | 1 (14%) |
| **Region** |  |  |  |
| East Anglia | 1 (2%) | 1 (3%) | 0 (0%) |
| East Midlands | 1 (2%) | 1 (3%) | 0 (0%) |
| London | 4 (10%) | 4 (11%) | 0 (0%) |
| North East | 6 (14%) | 0 (0%) | 6 (86%) |
| North West | 7 (17%) | 7 (20%) | 0 (0%) |
| Northern Ireland | 1 (2%) | 1 (3%) | 0 (0%) |
| Scotland | 5 (12%) | 5 (14%) | 0 (0%) |
| South East | 3 (7%) | 3 (9%) | 0 (0%) |
| South West | 3 (7%) | 3 (9%) | 0 (0%) |
| Wales | 3 (7%) | 3 (9%) | 0 (0%) |
| West Midlands | 5 (12%) | 5 (14%) | 0 (0%) |
| Yorkshire & Humberside | 3 (7%) | 2 (6%) | 1 (14%) |
| **Employment** |  |  |  |
| Paid Employment | 24 (57%) | 20 (57%) | 4 (57%) |
| Self-Employed | 2 (5%) | 2 (6%) | 0 (0%) |
| Unemployed | 2 (5%) | 2 (6%) | 0 (0%) |
| Full-Time Student | 2 (5%) | 1 (3%) | 1 (14%) |
| Looking After Home / Family | 2 (5%) | 2 (6%) | 0 (0%) |
| Retired | 8 (19%) | 7 (20%) | 1 (14%) |
| Other | 2 (5%) | 1 (3%) | 1 (14%) |
| **Highest Educational Qualification** |  |  |  |
| Degree or Equivalent | 26 (62%) | 22 (63%) | 4 (57%) |
| Higher Education Below Degree | 3 (7%) | 2 (6%) | 1 (14%) |
| A-Level/AS-Level | 4 (10%) | 4 (11%) | 0 (0%) |
| GCSE Grade A* - C | 4 (10%) | 4 (11%) | 0 (0%) |
| GCSE Grade D – G | 1 (2%) | 1 (3%) | 0 (0%) |
| Other | 1 (2%) | 0 (0%) | 1 (14%) |
| No Formal Qualification | 3 (7%) | 2 (6%) | 1 (14%) |
| **Weight Status** |  |  |  |
| Underweight | 2 (5%) | 2 (6%) | 0 (0%) |
| Normal/Healthy Weight | 25 (60%) | 19 (54%) | 6 (86%) |
| Overweight | 13 (31%) | 12 (34%) | 1 (14%) |
| Obese | 1 (2%) | 1 (3%) | 0 (0%) |
| Prefer Not To Say | 1 (2%) | 1 (3%) | 0 (0%) |
| **Easy to Understand the Questions** |  |  |  |
| Strongly Agree | 32 (76%) | 28 (80%) | 4 (57%) |
| Agree | 9 (21%) | 7 (20%) | 2 (29%) |
| Neither Agree or Disagree | 1 (2%) | 0 (0%) | 1 (14%) |
| Disagree | 0 (0%) | 0 (0%) | 0 (0%) |
| Strongly Disagree | 0 (0%) | 0 (0%) | 0 (0%) |
| **Easy to Tell the Difference between the Health States** |  |  |  |
| Strongly Agree | 24 (57%) | 21 (60%) | 3 (43%) |
| Agree | 16 (38%) | 13 (37%) | 3 (43%) |
| Neither Agree or Disagree | 2 (5%) | 1 (3%) | 1 (14%) |
| Disagree | 0 (0%) | 0 (0%) | 0 (0%) |
| Strongly Disagree | 0 (0%) | 0 (0%) | 0 (0%) |
| **Difficult to Decide on Answers** |  |  |  |
| Strongly Agree | 2 (5%) | 2 (6%) | 0 (0%) |
| Agree | 17 (40%) | 15 (43%) | 2 (29%) |
| Neither Agree or Disagree | 6 (14%) | 4 (11%) | 2 (29%) |
| Disagree | 13 (31%) | 10 (29%) | 3 (43%) |
| Strongly Disagree | 4 (10%) | 4 (11%) | 0 (0%) |

| **Table 2 – Responses to the WAItE** | |
| --- | --- |
|  | |
| **Mean WAItE Total Score (SD)** | **26.73 (5.27)** |
| **Median WAItE Total Score (IQR)** | **28 (23 – 31)** |
|  | |
| **WAItE Attributes & Levels** | **n (%)** |
| **I get tired** | |
| *Never* | 2 (5%) |
| *Almost Never* | 10 (24%) |
| *Sometimes* | 20 (48%) |
| *Often* | 8 (19%) |
| *Always* | 2 (5%) |
| **I struggle to keep up when walking around with others** | |
| *Never* | 21 (50%) |
| *Almost Never* | 8 (19%) |
| *Sometimes* | 6 (14%) |
| *Often* | 3 (7%) |
| *Always* | 4 (10%) |
| **I avoid doing sports** | |
| *Never* | 13 (31%) |
| *Almost Never* | 10 (24%) |
| *Sometimes* | 6 (14%) |
| *Often* | 4 (10%) |
| *Always* | 9 (21%) |
| **I struggle to concentration on my work/studies** | |
| *Never* | 8 (19%) |
| *Almost Never* | 13 (31%) |
| *Sometimes* | 17 (40%) |
| *Often* | 4 (10%) |
| *Always* | 0 (0%) |
| **I feel embarrassed shopping for clothes** | |
| *Never* | 28 (67%) |
| *Almost Never* | 9 (21%) |
| *Sometimes* | 1 (2%) |
| *Often* | 2 (5%) |
| *Always* | 2 (5%) |
| **I feel unhappy because I am unable to do the same things as others** | |
| *Never* | 20 (48%) |
| *Almost Never* | 7 (17%) |
| *Sometimes* | 9 (21%) |
| *Often* | 6 (14%) |
| *Always* | 0 (0%) |
| **People treat me differently when I go out** | |
| *Never* | 25 (60%) |
| *Almost Never* | 11 (26%) |
| *Sometimes* | 5 (14%) |
| *Often* | 0 (0%) |
| *Always* | 0 (0%) |

| **Table 3 – TTO and VAS Values for WAItE Health States (N=42)** | | | | |
| --- | --- | --- | --- | --- |
| **Health State** | **Health State A (2212122)** | **Health State B (2234442)** | **Health State C (4445555)** | **Pits State (5555555)** |
| TTO Mean (SD) | 0.95 (0.09) | 0.79 (0.19) | 0.39 (0.48) | 0.23 (0.54) |
| TTO Median (IQR) | 1 (0.95 – 1) | 0.80 (0.70 – 0.95) | 0.50 (0.20 – 0.70) | 0.33 (0.05 – 0.60) |
| Valuing State WTD (%) | 0 (0%) | 0 (0%) | 4 (10%) | 7 (17%) |
| VAS Mean (SD) | 84.48 (11.39) | 59.31 (12.89) | 28.45 (15) | 11.50 (11.78) |
| VAS Median (IQR) | 87.50 (80 – 90) | 60 (50 – 65) | 25.50 (20 – 40) | 10 (0 – 20) |
