# Supplementary material for: Valuing selected WAItE health states using the Time Trade-Off methodology: findings from an online interviewer-assisted remote survey
Source: J Patient Rep Outcomes. 2024 Jan 12;8:6. doi: 10.1186/s41687-023-00674-9 (PMC10786771; doi:10.1186/s41687-023-00674-9)
Supplement: Supplementary file 6 — Supplementary Material 6 [file 41687_2023_674_MOESM6_ESM.docx]

| **Reviewer 1** | |
| --- | --- |
| **Comment** | **Reply** |
| Thank you to the reviewers for their detailed and thoughtful comments, we feel as though they have greatly increased the quality of the manuscript. We have integrated the suggestions into the updated manuscript. Point by point responses to the comments are provided below. | |
| This study aims to obtain utility values for several health states from the Weight-Specific Adolescent Instrument for Economic Evaluation (WAItE) descriptive system from a sample of the UK adult general population. However, the WAItE was developed for adolescents and the cited literature demonstrated the validity of the instrument for use in the adolescent population. It is questionable that whether this instrument is applicable to adults. | The WAItE was developed for use in adolescence only. The adult sample is being used to generate a value set for the WAItE only, and the WAItE will not be used to evaluate HRQoL in the adult population.  We have updated the text in the first paragraph of the abstract (Page 2) and the second paragraph of the background section (page 3) and the first paragraph of the conclusion (Page 15) to emphasise this point further for the reader. |
| Also, the background section talks about the prevalence of obesity in adolescents only in the United Kingdom, which does not align with the research purposes. | As per the previous comment, the WAItE was developed for use in adolescence only. The adult sample is being used to generate a value set for the WAItE only, and the WAItE will not be used to evaluate HRQoL in the adult population.    Therefore, the introduction focusses specifically on the prevalence of obesity in adolescence and not the adult population. |
| As the authors stated, the TTO methodology is considered overly cognitively demanding for adolescents and there is an ethical concern with involving consideration of death with children and adolescents. I understand why the study targets on the adult population. However, more evidence is needed to demonstrate that this instrument is age-appropriate for the adult population. | As per the two previous comments, the WAItE was developed for use in adolescence only. The adult sample is being used to generate a value set for the WAItE only, and the WAItE will not be used to evaluate HRQoL in the adult population.  We have updated the text in the first paragraph of the abstract (Page 2) and the second paragraph of the background section (page 3) and the first paragraph of the conclusion (Page 15) to emphasise this point further for the reader. |
| The authors used videoconferencing software to deliver interviews. During the interview, subjects were asked to score each of the WAItE health states on a scale from 0 ('the worst health you can imagine') to 100 ('the best health you can imagine') using a visual analogue scale (VAS). One concern with the VAS using electronic devices is that photocopying or electronic rendering on different monitors or devices (e.g., tablet, smart phone, laptop) lead to different lengths, which can impact subjects' responses and thus makes the results hard to interpret. I recommend that the authors clarify whether any efforts were made to address this issue. If not, a discussion on the limitation of the VAS is needed. | The interviews all took place over videoconferencing software with the interviewee using a tablet or a laptop/desktop computer. It is possible that the VAS will have been a different length for different respondents depending on the size of their screen.  We have noted this as an additional limitation in the fifth paragraph of the ‘Strengths and Limitations’ section (Page 14). |
| The authors made the conclusions that "it is feasible to collect TTO data to an appropriate standard using digital interviews." and "all participants who started the online TTO interview fully completed the interview, indicating that the online interview, and interview process more generally, was fit for purpose." Please note that the quality of completion only cannot demonstrate whether the interview process is fit-for-purpose. The WAItE was developed as a Patient-Reported Outcome that should be completed by subjects themselves.  It is a concern that interviewers' description and interpretation of the questions may influence subjects' responses and leads to low test-retest reliability. Therefore, trainings for interviewers are very important to make sure the interview process is standardized. I recommend that the authors provide more information about what they did to address this concern and talk about relevant limitations in the article. | We agree with the reviewer that fully completing the interview is not the only marker of whether the interview process was fit for purpose. It is worth noting that the logical results also point to the interview process was fit for purpose, we have added another sentence to the first paragraph to the ‘Strengths and Limitations’ paragraph (Page 14) to emphasise this point.  As noted in the final paragraph of the ‘Piloting’ section (Page 8), the interviewers also were all using the same script. We have added an extra sentence to the first paragraph of the ‘Interview Procedure’ paragraph (Page 9) to emphasise this point.  In terms of test re-test reliability, it is worth noting that Oluboyede *et al* (2019) has shown that the WAItE has previously been found to have good levels of test-retest reliability. This is stated in the third paragraph of the background section (Page 3). |

| **Reviewer 2** | |
| --- | --- |
| **Comment** | **Reply** |
| This paper describes a time trade-off study that obtained valuations of a few of the health states in the Weight-Specific Adolescent Instrument for Economic Evaluation (WAItE) instrument, primarily to obtain values for the worst possible state in the measure for use in a larger valuation study. The rationale for the study is well articulated and the TTO and other methods are appropriate and clearly described. The study provides additional evidence that TTO interviews can be conducted virtually, using a video-conference platform. | Thank you. |
| The title of the paper seems broader than the aims of the study. The study did not obtain valuations for all of the WAItE states; the main goal was to get a value for the "pits state" for use in a larger study that will value all of the states. | We have updated the title to “Valuing Selected WAItE Health States using the Time Trade-Off Methodology: Findings from an Online Interviewer-Assisted Remote Survey”. |
| In the Abstract, it is not sufficiently clear what "weight" refers to. The introduction to the paper makes it clear that it refers to physical weight, related to overweight and obesity, but that is not clear in the abstract and the term could refer to some kind of methodological weighting process. | We have changed “weight” to “physical weight” in the abstract. |
| Throughout the paper, including in the Abstract, considerable clarification is needed about the use of adult respondents to provide valuations for a measure that is specific to adolescents. On page 6, it is noted that the TTO is to cognitively demanding for children and adolescents and that there are concerns about discussing death with young people. However, it is not clear whether the goal is to truly capture the preferences/utilities of adolescents. If that is the case, it seems essential for some work to be done with adolescents, possibly rating scale or VAS responses could be used instead of TTO. Alternatively, it might make sense to focus on young adults, whose views could be expected to be similar to adolescents. If a representative population sample was chosen to obtain a policy-relevant perspective, that should be explained. | The goal of the full valuation study (of which this TTO study is a part) is to generate a UK value set for the WAItE, which is a measure specific to adolescents. As the reviewer points out, throughout the paper we justify our use of an adult sample on several grounds, including the fact that TTO methods are unsuitable for use by adolescents.  We have added some extra text in the abstract (Page 1) clarifying that the WAItE is for use in adolescence and that adults completed the TTO survey.  We have added some extra text to the fifth paragraph of the ‘Background’ section further justifying the choice of an adult sample.  We have also added a further paragraph in the ‘Strengths and Limitations’ section (Page 15) acknowledging that the adult valuations may be different to those from adolescents and that this is an area of planned future research. |
| "Adolescent" should be clearly defined in terms of an age range. | We have included an extra sentence in the second paragraph of the background section (Page 3) clarifying that the age range for adolescence is 11-18. |
| The "pits state" is usually not capitalized as "PITS." It is not an acronym. This is a standard term in health utility assessment, but would merit definition early in the paper. | We have changed ‘PITS State’ to ‘Pits State’ throughout the manuscript. |
| It is not clear why there is reference to a "patient" information sheet on page 9 because the participants were not selected because they are patients. | We have updated the text in the ‘Interview Procedure’ paragraph (Page 9) from “Patient Information Sheet” to “Participant Information Sheet”. This was a typo – thank you to the reviewer for pointing this out. |
